# Long non-coding RNA ATB promotes malignancy of esophageal squamous cell carcinoma by regulating miR-200b/Kindlin-2 axis

**DOI:** 10.1038/cddis.2017.245

**Published:** 2017-06-22

**Authors:** Zhongwen Li, Xiaoliang Wu, Ling Gu, Qi Shen, Wen Luo, Chuangzhong Deng, Qianghua Zhou, Xinru Chen, Yanjie Li, ZuanFu Lim, Xing Wang, Jiahong Wang, Xianzi Yang

**Affiliations:** 1 Department of Oncology, Affiliated Hospital of Zunyi Medical University, Zunyi, China; 2 Department of Oncology, Guizhou Provincial People’s Hospital, Guiyang, China; 3 State Key Laboratory of Oncology in South China, and Collaborative Innovation Center for Cancer Medicine, Sun Yat-sen University Cancer Center, Guangzhou, China; 4 Puer University, Puer, China; 5 The 3rd Affiliated Hospital of Sun Yat-sen University, Guangzhou, China; 6 WVU Cancer Institute, Mary Babb Randolph Cancer Institute, Robert C. Byrd Health Sciences Center, West Virginia University, Morgantown, WV, USA; 7 Affiliated Cancer Hospital and Institute of Guangzhou Medical University, Guangzhou, China

## Abstract

Esophageal squamous cell carcinoma (ESCC) is one of the leading causes of cancer-related death, especially in China. In addition, the prognosis of late stage patients is extremely poor. However, the biological significance of the long non-coding RNA lnc-ATB and its potential role in ESCC remain to be documented. In this study, we investigated the role of lnc-ATB and the underlying mechanism promoting its oncogenic activity in ESCC. Expression of lnc-ATB was higher in ESCC tissues and cell lines than that in normal counterparts. Upregulated lnc-ATB served as an independent prognosis predictor of ESCC patients. Moreover, loss-of-function assays in ESCC cells showed that knockdown of lnc-ATB inhibited cell proliferation and migration both *in vitro* and *in vivo*. Mechanistic investigation indicated that lnc-ATB exerted oncogenic activities via regulating Kindlin-2, as the anti-migration role of lnc-ATB silence was attenuated by ectopic expression of Kindlin-2. Further analysis showed that lnc-ATB functions as a molecular sponge for miR-200b and Kindlin-2. Dysregulated miR-200b/Kindlin-2 signaling mediated the oncogenic activity of lnc-ATB in ESCC. Our results suggest that lnc-ATB predicts poor prognosis and may serve as a potential therapeutic target for ESCC patients.

Esophageal cancer has the eighth highest incidence rate among all cancer types worldwide, with varying occurrences from country to country.^1, 2^ China is one of those countries with highest risk of esophageal cancer.^3^ Esophageal squamous cell carcinoma (ESCC) arising from the squamous epithelium accounts for the most prevalent histological subtype.^2^ Despite recent advances in diagnosis technologies such as endoscopic ultrasonography and wide application of chemoradiotherapy combined with esophagogastric surgery in ESCC,^4, 5^ the overall survival of stage III patients is approximately 10–15% and <12 months for late stage patients.^6^ Therefore, new molecular biomarkers associated with tumor development and prognosis prediction are urgently needed in order to improve the understanding and outcome of this lethal disease.

Cancer is fundamentally regarded as a genetic disease^7^ that alters cellular information flow leading to uncontrolled proliferation and re-balanced cellular homeostasis.^8, 9^ Recent multiple genome-wide cancer studies have revealed an extensive landscape of long non-coding RNAs (lncRNAs) with exquisite regulation of the malignant transformation via interaction with other cellular macromolecules.^9, 10^ LncRNAs are a group of transcripts longer than 200 nucleotides from non-protein coding regions of the genome, the number of which is estimated to outnumber that of the protein coding genes.^11^ It is now recognized that lncRNAs may act as molecular sponge and competitively suppress expression of microRNAs (miRNAs).^10, 11^ One such lncRNA is the lncRNA activated by transforming growth factor *β* (TGF-*β*) (lnc-ATB), which was located in human chromosome 14 with 2446 nucleotides in length (Supplementary Table 1). Lnc-ATB contains multiple miR-200-binding sites and is known to be the mediator for TGF-*β*-induced epithelial–mesenchymal transition (EMT) in hepatocellular carcinoma.^12^ Moreover, lnc-ATB could promote trastuzumab resistance and the invasion-metastasis cascade in breast cancer by competitively binding miR-200c and upregulating ZEB1 and ZNF-217, resulting in EMT.^13^ Dysregulation of lnc-ATB have been reported in human tumors including gastric,^14^ colorectal,^15, 16^ brain,^17^ pancreatic^18^ and renal^19^ cancer. However, expression of lnc-ATB in ESCC and the underlying mechanisms remain unsolved.

## Results

### Lnc-ATB is upregulated in ESCC cancer tissues and cell lines and predicts unfavorable prognosis of ESCC patients

**Figure 1:**
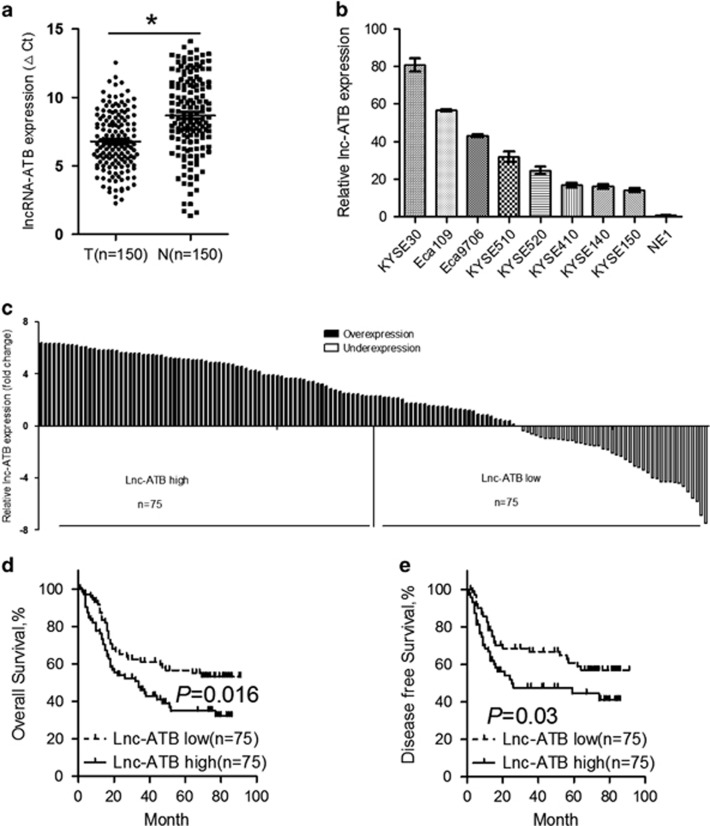
Lnc-ATB is upregulated in ESCC cancer tissues and cell lines and predicts poor prognosis. (**a**) Expression of lnc-ATB in 150 paired ESCC cancer tissues and adjacent normal tissues was detected by qRT-PCR and was normalized to that of GAPDH. Expression level of lnc-ATB was presented as △cycle threshold (△Ct) in tumor tissues relative to normal tissues. (**b**) Expression of lnc-ATB in ESCC cancer cells including KYSE30, Eca109, Eca9706, KYSE510, KYSE520, KYSE410, KYSE140 and KYSE150 and the normal epithelial cell line NE1 was detected by qRT-PCR. Expression level of lnc-ATB was analyzed with the 2^−△△Ct^ method and normalized to that of NE1. (**c**) Expression of lnc-ATB in 150 ESCC cancer tissues was detected by qRT-PCR. Expression level of lnc-ATB was presented as log_2_ fold change of △Ct value in tumor tissues to that of corresponding normal tissues. ESCC patients were divided into high lnc-ATB group (*n*=75) and low lnc-ATB group (*n*=75) according to the median value. (**d**) Kaplan–Meier curves of overall survival based on lnc-ATB expression in all 150 patients (*P*=0.016, log-rank test). (**e**) Kaplan–Meier curves of disease-free survival based on lnc-ATB expression in all 150 patients (*P*=0.03, log-rank test). For all quantitative results, the data are presented as the mean±S.E.M. from three independent experiments. *P<0.05

To define the role of lnc-ATB in ESCC, we examined the expression level of Lnc-ATB in 150 paired ESCC cancer and normal squamous epithelial tissues by qRT-PCR. We found that lnc-ATB was significantly upregulated in ESCC cancer tissues compared with the matched adjacent normal tissues (Figure 1a). Furthermore, expression of Lnc-ATB was also determined in a panel ESCC cancer cell lines (KYSE30, Eca109, Eca9706, KYSE510, KYSE520, KYSE410, KYSE140 and KYSE150) and one immortalized normal epithelial cells (NE1). The results showed that lnc-ATB expression was upregulated in the ESCC cancer cell lines when compared with NE1 cells (Figure 1b). We then analyzed the correlation between Lnc-ATB expression and the clinicopathological features of 150 ESCC patients. Using the median expression level as the cutoff value, our patient cohort was assigned into high lnc-ATB expression group (above the median, *n*=75) and low lnc-ATB expression group (below the median, *n*=75) (Figure 1c). The correlations between expression of Lnc-ATB and the clinical pathological parameters of ESCC patients are summarized in Table 1.

To further analyze whether lnc-ATB expression could be a potential prognosis predictor in ESCC patients, we performed a Kaplan–Meier analysis with log-rank test. The results showed that the median overall survival of patients with high lnc-ATB expression was 20 months, whereas the median overall survival for patients with low lnc-ATB expression was 51 months (Figure 1d). Moreover, expression of lncRNA correlates with disease-free survival of ESCC patients (*P*=0.03, Figure 1e). Further univariate and multivariate survival analyses showed that TNM stage (*P*<0.001) and Lnc-ATB expression (*P*=0.023) were independent prognosis indicators of ESCC patients (Table 2). Collectively, lnc-ATB is upregulated in ESCC and predicts poor prognosis of patients.

### Knockdown of lnc-ATB inhibits proliferation and induces cell cycle arrest of ESCC cells

**Figure 2:**
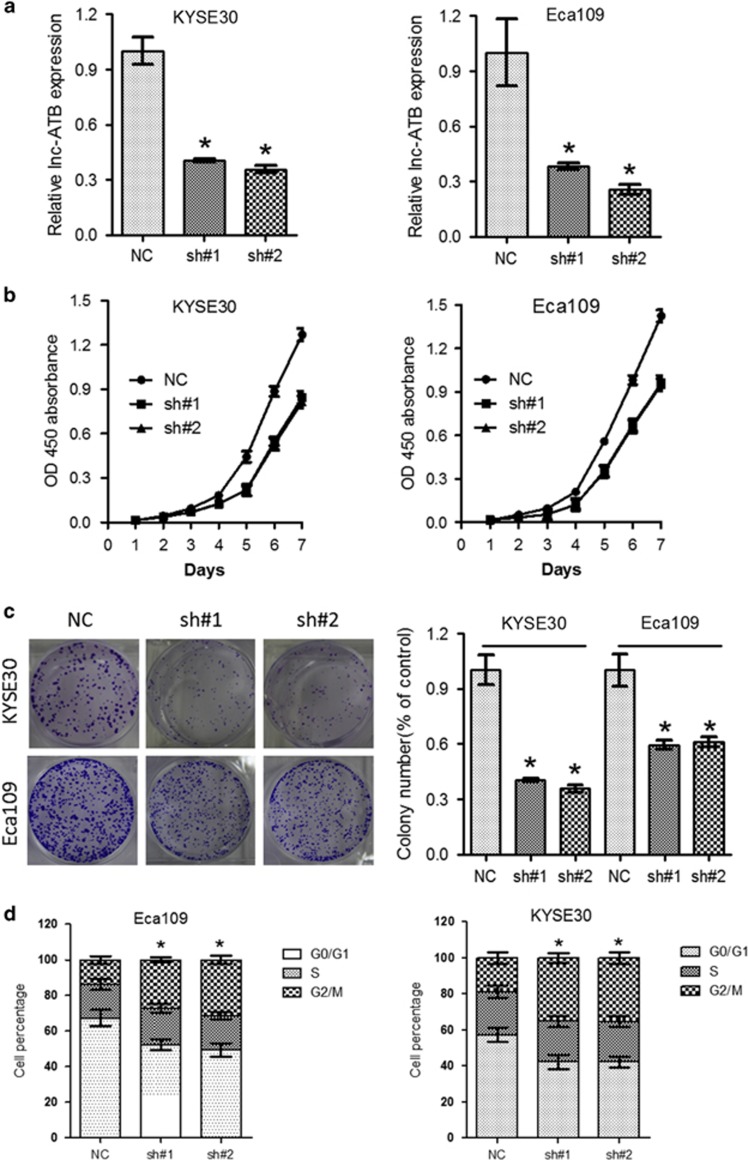
Knockdown of Lnc-ATB inhibits proliferation and induces cell cycle arrest of ESCC cells. (**a**) Expression of lnc-ATB was detected in KYSE30 and Eca109 cells after treatment with indicated short hairpin RNAs, whereas sh#1 and sh#2 indicates two independent oligonucleotides targeting lnc-ATB. (**b**) CCK-8 assays of KYSE30 and Eca109 cells after knockdown of lnc-ATB. (**c**) Colony formation assays of KYSE30 and Eca109 cells after knockdown of lnc-ATB. (**d**) Cell cycle assays of KYSE30 and Eca109 cells after knockdown of lnc-ATB. For all quantitative results, the data are presented as the mean±S.E.M. from three independent experiments. *P<0.05

To explore the functional relevance of dysregulated lnc-ATB in ESCC, KYSE30 and Eca109 cells were selected for further analysis. Isolation of cytoplasmic and nuclear RNAs indicated that lnc-ATB was mainly located in the cytoplasm (Supplementary Figure 1). Lentivirus stably expressing short hairpin RNAs targeting lnc-ATB (sh#1 and sh#2), as well as the scramble sequence (NC) were introduced into ESCC cells and qRT-PCR analysis confirmed effective knockdown of lnc-ATB in KYSE30 and Eca109 cells (Figure 2a). CCK-8 assays revealed that knockdown of lnc-ATB significantly decreased cell viability of ESCC cell lines (KYSE30 and Eca109) (Figure 2b). On the other hand, KYSE30 and Eca109 cells with relative high lnc-ATB expression showed faster proliferation rate than that of KYSE140 and KYSE410 cells with low lnc-ATB expression (Supplementary Figure 2A). Moreover, knockdown of lnc-ATB significantly suppressed formation of colonies compared with sh-control group in KYSE30 and Eca109 cells (Figure 2c). We then investigated whether the anti-proliferation roles of lnc-ATB knockdown was associated with cell cycle arrest or increased apoptosis. To this end, KYSE30 and Eca109 cells (sh#1, sh#2 and negative control (NC)) were subjected to cell cycle and cell apoptosis analysis by flow cytometry. As shown in Figure 2d and Supplementary Figure 2B, knockdown of lnc-ATB significantly induced G2/M phase arrest and apoptosis in KYSE30 and Eca109 cells.

### Knockdown of lnc-ATB inhibits migration and lung metastasis of ESCC cells

**Figure 3:**
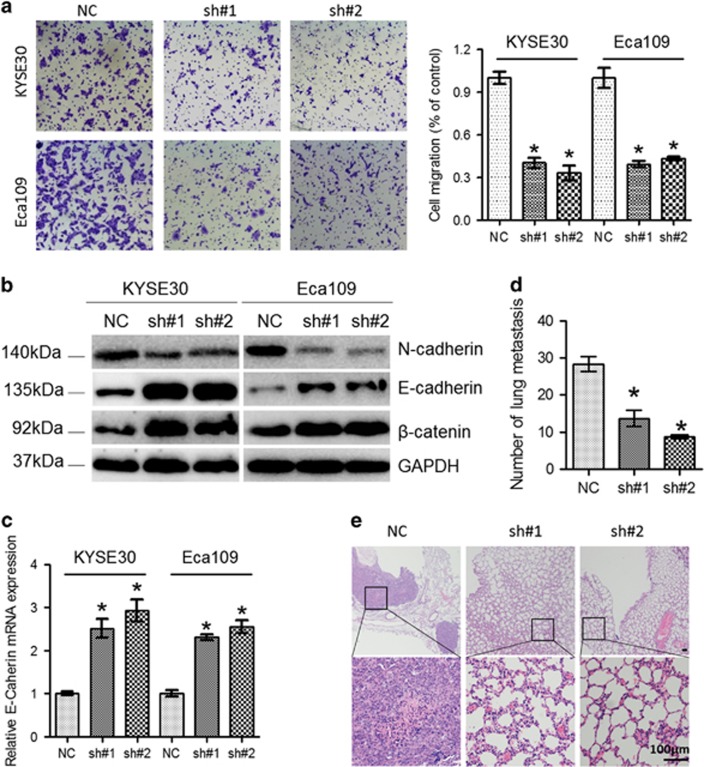
Knockdown of Lnc-ATB decreased migration and suppressed lung metastasis of ESCC. (**a**) The representative pictures and quantification of cell migration across the membrane in KYSE30 and Eca109 cells with lnc-ATB reduction. (**b**) The protein expression of E-Cadherin, N-Cadherin and *β*-Catenin in KYSE30 and Eca109 cells with stable lnc-ATB reduction were evaluated by western blotting. GAPDH was used for normalization. (**c**) mRNA level of E-Cadherin in KYSE30 and Eca109 cells with stable lnc-ATB reduction was detected. (**d**) The nodules in the lung were numbered (*n*=6). (**e**) Visualization of HE-stained lung sections are shown. Scale bars: 100 *μ*m. For all quantitative results, the data are presented as the mean±S.E.M. from three independent experiments. *P<0.05

We then sought to investigate migratory and invasive capacity of ESCC cells after knockdown of lnc-ATB. ESCC cells with lnc-ATB reduction had a significant decrease in cell migration (Figure 3a). EMT has vital roles in cancer metastasis.^20^ Western blot analysis revealed that knockdown of lnc-ATB led to elevated expression of the epithelial markers including E-cadherin and *β*-catenin and decreased expression of the mesenchymal marker N-cadherin (Figures 3b and c), indicating that EMT may be associated with the pro-metastasis effects of lnc-ATB in ESCC. We then injected Eca109 cells with lnc-ATB suppression into the tail vein of nude mice and found that the metastatic nodules in the lung were significantly decreased in the knockdown group compared with that in the control group (Figures 3d and e).

### Correlation of expression of Lnc-ATB with miR-200b

**Figure 4:**
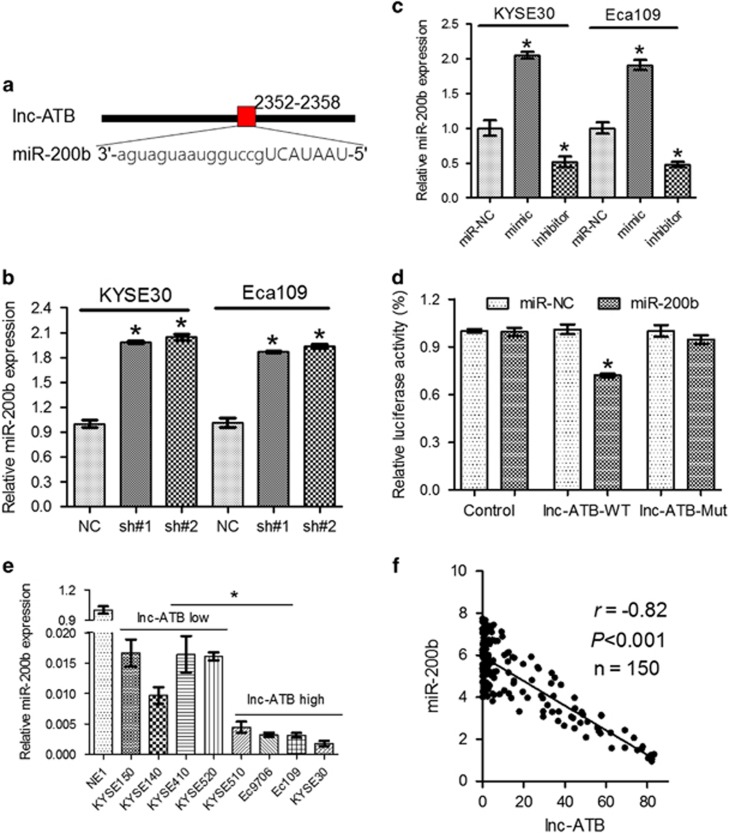
Correlation between expression levels of Lnc-ATB and miR-200b. (**a**) Diagram showing binding site in lnc-ATB of miR-200b. (**b**) Expression of miR-200b in KYSE30 and Eca109 cells after transfection with mimics or inhibitor. (**c**) Expression of miR-200b in KYSE30 and Eca109 cells with lnc-ATB reduction. (**d**) Luciferase assays of KYSE30 and Eca109 cells after transfection with WT or mutant reporter vector and miR-200b mimic. (**e**) Expression of miR-200b in the ESCC cancer cell lines. (**f**) Expression of miR-200b of lnc-ATB in the ESCC tumor samples. For all quantitative results, the data are presented as the mean±S.E.M. from three independent experiments. *P<0.05

Previous studies have reported that many lncRNAs function as a competing endogenous RNAs (ceRNA) to bind disease specific miRNAs such as the case of lnc-ATB and miR-200 family in hepatocellular carcinoma^12^ and glioma.^17^ To examine whether lnc-ATB acts in a similar manner, prediction of miRNA target sites in starbase (http://starbase.sysu.edu.cn/) was performed. We found multiple miR-200b-binding sites in lnc-ATB (Figure 4a) and further RNA immunoprecipitation (RIP) analysis confirmed that miR-200b was significantly enriched with lnc-ATB (Supplementary Figure 3). Moreover, tumor-suppressive roles of miR-200b have been reported in several human cancers including ESCC,^21, 22^ so we focused on miR-200b as the primary candidate. We found that expression of miR-200b was remarkably increased after knockdown of lnc-ATB (Figure 4b). Next, we constructed luciferase reporters, which contain wild-type (WT) or mutated (Mut) miR-200b-binding sites, for direct binding validations (Figure 4c). As shown in Figure 4d, miR-200b mimics significantly reduced the luciferase activities of the WT reporter vector but did not affect the activity of the mutant vector compared with miR-NC, supporting the notion that miR-200b bind bona fide to lnc-ATB. Moreover, miR-200b was significantly upregulated in ESCC cell lines with relative low lnc-ATB expression than cell lines with high expression (Figure 4e). Expression of miR-200b and lnc-ATB showed an inverse correlation pattern in our patient cohort (Figure 4f), providing clues that lnc-ATB may function as ceRNA for miR-200b.

### Modulation of Kindlin-2 by lnc-ATB

**Figure 5:**
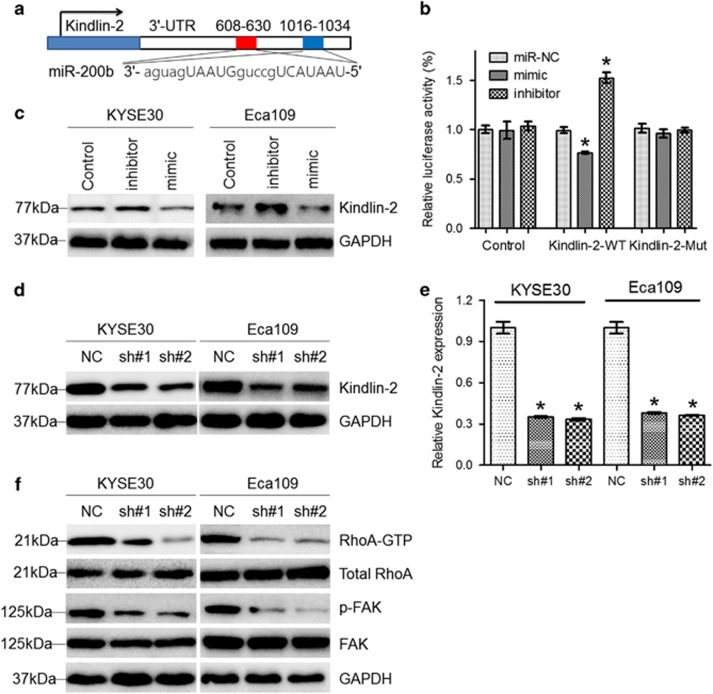
Lnc-ATB regulates expression of Kindlin-2. (**a**) Diagram showing binding site in Kindlin-2 of miR-200b. (**b**) Luciferase assays in cells transfected with WT or mutant 3′-UTR reporter vector and miR-200b mimic or inhibitor. (**c**) Immunoblotting of Kindlin-2 in KYSE30 and Eca109 cells transfected with miR-200b mimic or inhibitor. GAPDH was used for normalization. (**d**) Immunoblotting of Kindlin-2 in KYSE30 and Eca109 cells with lnc-ATB reduction. GAPDH was used for normalization. (**e**) Relative mRNA level of Kindlin-2 in KYSE30 and Eca109 cells with lnc-ATB reduction. (**f**) Immunoblotting of RhoA-GTP and p-FAK in KYSE30 and Eca109 cells following lnc-ATB reduction. GAPDH was used for normalization. For all quantitative results, the data are presented as the mean±S.E.M. from three independent experiments. *P<0.05

Decreased expression of miR-200b was reported to predict poor prognosis of ESCC patients and create malignant phenotype via modulation of Kindlin-2.^21, 22^ We found that miR-200b could bind to the 3′-UTR of Kindlin-2 and modulate expression of Kindlin-2 in KYSE30 and Eca109 cells (Figures 5a–c). Moreover, expression of Kindlin-2 was suppressed in KYSE30 and Eca109 cells after knockdown of lnc-ATB (Figure 5d). qRT-PCR analysis indicated that mRNA level of Kindlin-2 was decreased by lnc-ATB knockdown (Figure 5e). Kindlin-2 was previously reported to be involved in the cytoskeleton shaping via RhoA/FAK signaling. Immunoblotting showed that active RhoA and phosphorylated FAK was suppressed after knockdown of lnc-ATB (Figure 5f).

### miR-200b/Kindlin-2 mediates oncogenic activity of lnc-ATB

**Figure 6:**
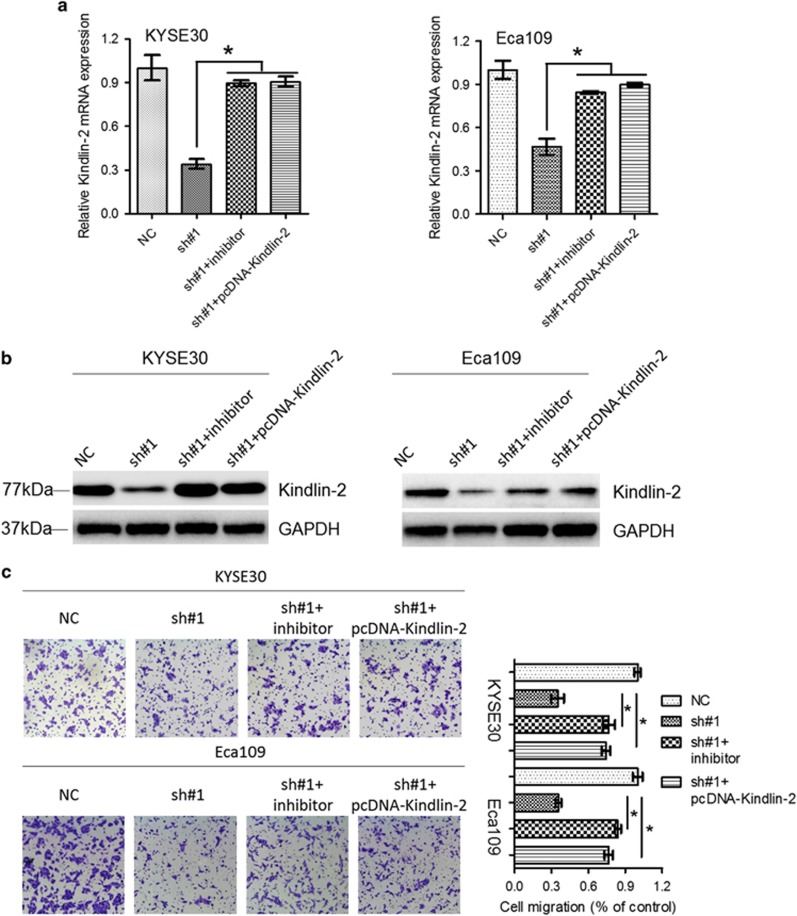
Kindlin-2 mediates the oncogenic activity of lnc-ATB. (**a**) Relative mRNA level of Kindlin-2 in KYSE30 and Eca109 cells following lnc-ATB suppression and transfection with miR-200b inhibitor or a pcDNA-Kindlin-2 lacking the 3′-UTR. (**b**) Immunoblotting of Kindlin-2 in KYSE30 and Eca109 cells following lnc-ATB suppression and transfection with miR-200b inhibitor or a pcDNA-Kindlin-2 lacking the 3′-UTR. GAPDH was used for normalization. (**c**) The representative pictures and quantification of cell migration across the membrane in KYSE30 and Eca109 cells following lnc-ATB suppression and transfection with miR-200b inhibitor or a pcDNA-Kindlin-2 lacking the 3′-UTR. For all quantitative results, the data are presented as the mean±S.E.M. from three independent experiments. *P<0.05

To determine whether miR-200b/Kindlin-2 mediated oncogenic effects of lnc-ATB in ESCC, KYSE30 and Eca109 cells with stable lnc-ATB reduction were transfected with miR-200b inhibitors or a vector with the coding sequence of Kindlin-2 lacking the 3′-UTR (pcDNA-Kindlin-2). We found that miR-200b inhibitors or pcDNA-Kindlin-2 significantly increased mRNA and protein levels of Kindlin-2 (Figures 6a and b). Moreover, attenuation of Kindlin-2 significantly reversed the anti-migration roles of lnc-ATB knockdown (Figure 6c).

### Knockdown of Lnc-ATB significantly suppressed tumor growth *in vivo*

**Figure 7:**
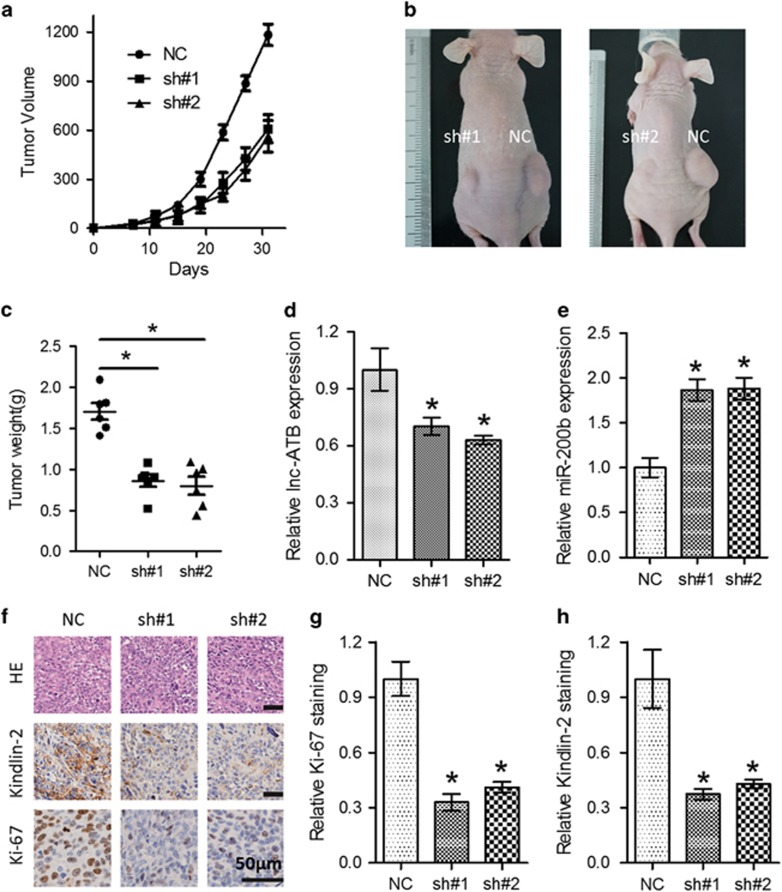
Lnc-ATB suppresses tumor growth via miR-200b/Kindlin-2 *in vivo*. (**a**) Tumor growth curve formed by the indicated cells. (**b**) Tumors formed in the nude mice were photographed. (**c**) Tumor weight of mice in the NC, sh#1 and sh#2 groups were recorded. The expression levels of lnc-ATB (**d**) and miR-200b (**e**) in the dissected tumors were measured. (**f**) Immunohistochemical staining of Ki-67 and Kindlin-2 in the xenograft tumors. Scale bars: 50 *μ*m. (**g**) Quantitative analysis for immunostaining of Ki-67 (**g**) and Kindlin-2 (**h**) in the xenograft tumors. For all quantitative results, the data are presented as the mean±S.E.M. from three independent experiments. *P<0.05

To investigate whether lnc-ATB regulates ESCC tumorigenesis *in vivo*, Eca109 cells (sh#1, sh#2 and NC) were subcutaneously injected into the dorsal flank of female nude mice and the tumor volume was monitored. As shown in Figures 7a–c, the volume and weight of xenografts in the knockdown groups were significantly smaller compared with those formed in NC group, which is consistent with *in vitro* results. Tumor weight was significantly reduced in the knockdown group than that in the control group (Figure 7c). Moreover, qRT-PCR analysis of the dissected xenografts from the knockdown group showed decreased expression of lnc-ATB and increased expression of miR-200b than that from control group (Figures 7d and e). Immunostaining of Ki-67 and Kindlin-2 in dissected tumors showed that silencing of lnc-ATB inhibited proliferation and Kindlin-2 of ESCC cells (Figures 7f–h), indicating that knockdown of lnc-ATB significantly suppressed tumor growth via miR-200b/Kindlin-2 *in vivo.*

## Discussion

ESCC constitutes the main histopathological subtype of esophageal cancer occurring in the central regions of China.^3^ The overall prognosis of ESCC remains unsatisfactory despite recent progression in diagnostic methods and therapeutic alternatives.^1, 2, 4, 5^ To improve the survival rate of ESCC patients, a better understanding of the genetic and/or epigenetic alterations underlying tumorigenesis and metastasis of ESCC is urgently required. Dysregulation of lncRNAs has been previously reported in a wide range of human cancers including ESCC.^10, 11, 14, 17, 19, 23, 24, 25^ For example, the lncRNA MALAT1,^23, 25^ HOTTIP^26^ and HOTAIR^27^ were overexpressed in ESCC tumor tissues and are good predictive factors for overall survival. Moreover, increased expression of lnc-ATB was observed in breast cancer,^13^ gastric cancer^14^ and colorectal cancer,^15, 16^ indicating that lnc-ATB may function as oncogenes in these cancers. Inspired by these observations, we sought to determine the role of lnc-ATB in ESCC. We were able to detect lnc-ATB in 150 ESCC tumor tissues and paired normal counterparts and draw correlations between the expression of lnc-ATB with clinicopathological parameters. Our data revealed that lnc-ATB was significantly upregulated in ESCC tumor tissues and patients with high lnc-ATB expression showed poorer prognosis than those with low expression. Expression of lnc-ATB was associated with tumor invasion, lymph node metastasis and differentiation state in ESCC. This was validated in our current study, in which elevated lnc-ATB expression was positively correlated with ESCC carcinogenesis. However, Qu *et al.*^18^ reported that expression of lnc-ATB decreased in pancreatic cancer tissues and cell lines, and decreased lnc-ATB expression was an independent predictor of poor prognosis in pancreatic cancer patients. Overall, these data suggest that there is a cancer specific expression pattern of lnc-ATB.

Previous studies have indicated that knockdown of lnc-ATB inhibited EMT and cell invasion in hepatocellular carcinoma^12^ and colorectal cancer^16^ *in vitro* as well as tumor growth in the nude mice. We therefore proceeded to evaluate the biological roles of lnc-ATB in ESCC by short hairpin RNA-mediated loss-of function assays. Silencing of lnc-ATB expression significantly suppressed proliferation and migration of ESCC cells compared with control. EMT is a critical process associated with tumor progression and metastasis. Our data showed that knockdown of lnc-ATB downregulated Vimentin and N-cadherin and upregulated E-cadherin. These findings demonstrate that lnc-ATB may have a role in EMT.

Tumor-suppressive effects of miR-200 family has been repeatedly verified during the past few years,^21, 22, 28, 29^ whereby miR-200c initiates malignant transformation by modulation of cancer stem cells,^30^ and decreased miR-200 promotes distant metastasis via regulation of the EMT activator ZEB1 and ZEB2.^31^ Moreover, chr1p36 containing the miR-200b cluster is often deleted in ESCC.^32^ Overexpression of miR-200b induces cell cycle arrest and represses cell growth, migration and invasion of ESCC cells.^21, 22^ Our results showed that expression of miR-200b was significantly upregulated after knockdown of lnc-ATB, and further dual luciferase assays confirmed direct binding of lnc-ATB and miR-200b.

Cells with dynamic assembly of cytoskeleton and active focal adhesion with the surrounding microenvironment are predisposed to local invasion and distant metastasis.^33^ Kindlin-2, a FERM domain containing protein, has been shown to modulate cell migration via interactions with the cytoskeleton, resulting in cell–extracellular matrix adhesions in human cancers including ESCC.^21, 22, 34, 35, 36, 37^ Furthermore, overexpression of Kindlin-2 was observed in the invasive front of malignant mesothelioma^38^ and breast cancer,^35^ whereas knockdown of Kindlin-2 exerts tumor-suppressive roles. Upregulation of Kindlin-2 induced by decreased miR-200b promotes malignant phenotype of ESCC and predicts unfavorable prognosis.^21, 22^ In this study, we found that overexpression of lnc-ATB may function as molecular sponge of Kindlin-2 and miR-200b. Attenuation of the anti-proliferative roles of lnc-ATB knockdown by re-induction of Kindlin-2 in ESCC cells further demonstrated the role of the lnc-ATB/miR-200b/Kindlin-2 axis in tumor metastasis. Notably, this mechanism may correlate with our clinical observations that the loss of miR-200b in ESCC tumors is associated with lymph node metastasis, advanced clinical stage and short survival.

## Conclusions

Our current study demonstrates that upregulation of lnc-ATB is associated with ESCC progression. Our results provide new insights into the dysregulated lnc-ATB/miR-200b/Kindlin-2 axis in the development of ESCC and suggest that lnc-ATB represent a potential therapeutic target for ESCC.

## Materials and methods

### Patients and samples

The ESCC tumor tissues and corresponding normal esophageal epithelial tissues were obtained from 150 patients who underwent surgery at the Sun Yat-sen University Cancer Center from 2007 to 2009, Guangzhou, China. No patients enrolled in our study were given local or systemic treatment before surgery. Clinicopathological data were collected from medical records. All specimens were snap-frozen immediately after resection from the patient and stored at −80 °C until RNA extraction.

### Cell lines and cell culture

The human ESCC cell lines (KYSE150, KYSE140, KYSE410, KYSE520, KYSE510, Ec109, Ec9706 and KYSE30) were obtained either from the Cell Bank of the Chinese Academy of Sciences (Shanghai, China) or purchased from the Deutsche Sammlung von Mikroorganismen und Zellkulturen (DSMZ, Braunschweig, Germany). The human embryonic kidney cell line HEK293a were purchased from the American Type Culture Collection (Manassas, VA, USA). All cells were cultured in Dulbecco’s modified essential medium supplemented with 10% fetal bovine serum (FBS, Hyclone, Logan, UT, USA), 100 U/ml penicillin (Sigma-Aldrich, St. Louis, MO, USA) and 100 *μ*g/ml streptomycin (Sigma-Aldrich) and maintained in a humidified incubator at 37 °C with 5% CO_2_.

### RNA extraction and quantitative real-time PCR

Total RNAs from cell lines and tissue samples was extracted using Trizol reagent (Invitrogen, Carlsbad, CA, USA) according to the manufacturer’s protocol. Cytoplasmic and nuclear RNA were isolated with the commercial kit (NORGEN, Thorold, ON, Canada) according to the manufacturer’s instructions. RNA concentration and quality were evaluated by the 260/280 nm absorbance with a Nanodrop Spectrophotometer (IMPLEN GmbH, Munich, Germany). The RNA was then reversely transcribed to cDNA with the PrimeScript RT Master Mix (Takara Biotechnology, Dalian, China). SYBR Green Mix (Promega, Madison, WI, USA) was used to detect expression of lnc-ATB and Kindlin-2, whereas GAPDH was used as an internal control. The primer sequences were as follows:

lnc-ATB forward: 5′-TCTGGCTGAGGCTGGTTGAC-3′

lnc-ATB reverse: 5′-ATCTCTGGGTGCTGGTGAAGG-3′

Kindlin-2 forward: 5′- TGTCTCCCCGCTATCTAAAAAAGT-3′

Kindlin-2 reverse: 5′- TGATGGGCCTCCAAGATTCT-3′

E-Cadherin forward: 5′- CGAGAGCTACACGTTCACGG-3′

E-Cadherin forward: 5′- GGGTGTCGAGGGAAAAATAGG-3′

GAPDH forward: 5′- GCAAGAGCACAAGAGGAAGA-3′

GAPDH reverse: 5′-ACTGTGAGGAGGGGAGATTC-3′.

For measurement of miRNAs, TaqMan MicroRNA Assay kits (Applied Biosystems, Darmstadt, Germany) were used and real-time PCR reaction was carried out using ABI 7500 fast real-time PCR system (Applied Biosystems). U6 served as the internal control. PCR cycling conditions were 10 min at 95 °C followed by 45 cycles of 95 °C for 15 s and 60 °C for 60 s. All reactions were performed in triplicates. The fold change for each gene relative to the control group was calculated using the 2^−ΔΔCt^ method.

### Cell transfection

Lentivirus containing the short hairpin RNA targeting human lnc-ATB (Supplementary Table 2) was obtained from GenePharma (Shanghai, China), and was labeled as sh#1, sh#2 and its corresponding non-targeting sequence (sh#NC). The ESCC cells were transfected with the lentivirus before they were selected with puromycin (3 *μ*g/ml) for 72 h and the efficiency was confirmed by GFP-positive cells percentage. The miR-200b mimics, miR-200b inhibitors and miR-200b NC were obtained from Ribobio (Guangzhou, China), and transfected into the ESCC cell lines by using Lipofectamine2000 (Invitrogen) according to the manufacturer’s protocol.

### Cell proliferation, cell cycle and cell apoptosis assays

The proliferation of ESCC cells was detected by the Cell Counting Kit-8 (CCK-8, Beyotime Institute of Biotechnology, Jiangsu, China). Briefly, cells were placed into 96-well plates at the density of 1000 cells per well before addition of CCK-8 and the absorbance was measured at a wavelength of 450 nm. Five replicates were conducted for cells in each group and the experiments were repeated three times. For the clone formation assay, cells transfected with the indicated lentivirus were seeded in six-well plates in triplicates at a density of 500 cell per well and incubated at 37 °C with 5% FBS for 14 days. The cells were fixed with 4% polyoxymethylene and stained with 1.5% methylene blue. The colonies were counted and analyzed by Image J software (NIH, Bethesda, MD, USA). For cell cycle analysis, the indicated cells were collected, rinsed with cold PBS twice, and labeled with propidium iodide (PI) solution (BD Cycletest Plus DNA Kit, San Jose, CA, USA). For cell apoptosis analysis, Annexin V/PI kits (KeyGEN, Nanjing, China) were used to detect apoptotic cells. The cells were then analyzed by a fluorescence-activated cell sorter according to a previous report.^21^

### Cell migration assays

The Corning Polycarbonate Membrane Insert transwell chambers (Product #3422, Corning Costar Corp, Cambridge, MA, USA) were used to perform migration assays. Briefly, cells (1 × 10^5^) in serum-free media were placed into the upper chamber and medium containing 20% FBS functioning as a chemoattractant was placed in the lower chamber. After 36-h incubation, cells were fixed with 4% polyoxymethylene and stained with 1.5% methylene blue. The cells remaining in the upper chamber were removed with cotton swabs and those migrated or invaded cells were imaged and counted under the microscope. Experiments were independently repeated three times.

### Western blot analysis

Total lysates were obtained from harvested ESCC cells with the 1 × RIPA buffer (Cell Signaling Technology, Danvers, MA, USA) supplemented with protease inhibitors. The concentration was determined by the BCA Protein Assay kit (Pierce, Rockford, IL, USA) and identical quantities of proteins (20–30 *μ*g) were separated by SDS-polyacrylamide gel electrophoresis. The proteins were then transferred to PVDF membranes. After blocking with 5% non-fat milk in TBST, the membranes were probed with the indicated primary antibodies at 4 °C overnight with gentle shaking. The membranes were then exposed to the corresponding secondary antibody and the SupreSignal ECL Kit (Pierce) was used to detect bands, with GAPDH as the internal control. The primary antibodies used in our study include E-cadherin (1:500), Vimentin (1:500), *β*-catenin (1:500, Cell Signaling Technology), Kindlin-2 (1:2000, Millipore, Bedford, MA, USA) p-FAK(Tyr397) (1:1000, Cell Signaling Technology), FAK (1:500, Cell Signaling Technology), active RhoA (1:500, Cell Signaling Technology), total RhoA (1:500, Cell Signaling Technology) and GAPDH (1:5000, Sigma, Sigma-Aldrich, St. Louis, MO, USA). HRP-conjugated goat anti-rabbit or anti-mouse IgG antibody (Abcam, Cambridge, MA, USA) was used as the secondary antibody.

### Luciferase reporter assay

The cDNA encoding lnc-ATB was amplified by the Pfu Ultra II Fusion HS DNA Polymerase (Stratagene, Agilent Technologies, Santa Clara, CA, USA) and sub-cloned into the *Hind*III and *EcoR*I sites of pcDNA3.1 vector (Invitrogen), named pcDNA3.1-ATB-WT. The pcDNA3.1-ATB-Mut vector, which contains point mutations in the miR-200b response elements was generated with a Quick Change Site-Directed Mutagenesis kit (Stratagene, Agilent Technologies). The miR-200b targeting region at the 3′ end of either pcDNA3.1-ATB-WT or pcDNA3.1-ATB-Mut was PCR-amplified and sub-cloned into the pmir-GLO vector (Promega) using the one step directed cloning kit (Novoprotein, Shanghai, China), which were named as lncRNA-ATB-WT or lncRNA-ATB-Mut, respectively. The primers used for vector construction was provided in Supplementary Table 3. Dual luciferase reporter assays were performed by co-transfection in the indicated cell lines with pmir-GLO-lnc-ATB or pmir-GLO-lnc-ATB-Mut vectors, and miR-200b mimics or miR-NC. At 48 h post-transfection, the luciferase activities were measured with the Dual Luciferase Reporter Assay System (Promega) according to the manufacturer instructions. Data are presented as ratios between firefly and Renilla fluorescence activities. Each experiment were performed independently in triplicates.

### RIP assays

RIP assays were conducted with a Magna RNA-binding protein immunoprecipitation kit (Millipore) according to the manufacturer’s instructions. Briefly, cell lysates were incubated with RIP buffer containing magnetic beads conjugated with negative IgG or anti-Ago2 antibody. Immunoprecipitated RNAs were obtained by digestion with Proteinase K. Then, the extracted RNAs were reversely transcribed into complementary DNA and subjected to quantitative real-time PCR analysis.

### Animal work

Four-week female BALB/c mice were maintained in laminar airflow chambers under specific pathogen-free conditions. The indicated cells were suspended in cold PBS at a final concentration of 1 × 10^7^ cells/ml, whereas 100 *μ*l of suspended cells was subcutaneously injected into the right flank of each mouse (*n*=5 per group). The tumor volume was monitored every 2 days and calculated with the formula: 0.5 × length × width^2^. At 24 days post injection, the mice were killed by cervical dislocation. The dissected tumors were weighed and paraffin embedded for immunohistochemical analysis of Ki-67 expression. For the metastasis model, the Eca109 cells with lnc-ATB reduction were injected into the tail vein of nude mice (*n*=6 per group). Eight weeks post injection, the mice were killed and the lungs were removed and paraffin embedded. Consecutive sections (4 *μ*m) were made and stained with haematoxylin–eosin. The micrometastases in the lungs were examined and counted. This study was carried out strictly in accordance with the recommendations in the Guide for the Care and Use of Laboratory Animals of the National Institutes of Health. The experimental protocols were approved by the committee on animal experimentation of the Sun Yat-sen University Cancer Center.

### Statistical analysis

All statistical analyses were performed using the SPSS 19.0 statistical software package (SPSS, Chicago, IL, USA). The data are representative of three independent experiments and are presented as mean value±S.D. To evaluate significant differences between two independent groups of samples, Student’s *t*-test was used. The Kaplan–Meier method was used to generate the survival curve and compared using the log-rank test. The chi-square test or Fisher’s exact test was used to analyze the association of lnc-ATB expression with clinicopathological parameters. *P*-value <0.05 was considered to be statistically significant.

## Figures and Tables

**Table 1:** The correlation between clinicopathological parameters and lnc-ATB expression

	**Lnc-ATB expression**		
	**Low, *n*(%)**	**High, *n* (%)**	
*Age*			
<60	44 (58.7)	39 (52.0)	0.511
⩾60	31 (41.3)	36 (48.0)	
			
*Gender*			
Male	61 (81.3)	57 (76.0)	0.550
Female	14 (18.7)	18 (24.0)	
			
*Alcohol consumption*			
Ever and current	52 (69.3)	47 (62.7)	0.491
Never	23 (30.7)	28 (37.3)	
			
*Smoking status*			
Ever and current	44 (58.7)	43 (57.3)	1.000
Never	31 (41.3)	32 (42.7)	
			
*Tumor size*			
<5 cm	63 (84.0)	62 (82.7)	1.000
⩾5 cm	12 (16.0)	13 (17.3)	
			
*Differentiation status*			
Well or moderate	59 (78.7)	59 (78.7)	1.000
Poor	16 (21.3)	16 (21.3)	
			
*TNM stage*			0.514
I–II	41 (54.7)	36 (48.0)	
III	34 (45.3)	39 (52.0)	

**Table 2:** Univariate and multivariate analyses of various potential prognostic factors in ESCC patients

	**Univariate analysis**		**Multivariate analysis**	
	**HR (95% CI)**	***P*****-value**	**HR (95% CI)**	***P*****-value**
Age (<60 /⩾60)	1.43 (0.92–2.24)	0.112	—	—
Gender (male/female)	0.92 (0.54–1.56)	0.757	—	—
Alcohol (never/ever)	0.98 (0.61–1.58)	0.921	—	—
Smoke (never/ever)	0.96 (0.61–1.50)	0.842	—	—
Tumor size (⩾5 cm/<5 cm)	1.48 (0.84–2.60)	0.176	—	—
Differentiation (moderate/poor, well)	1.49 (0.90–2.47)	0.118	—	—
TNM stage (III/I–II)	3.65 (2.25–5.92)	0.000	3.59 (2.21–5.83)	0.000
lnc-ATB (high/low)	1.76 (1.22–2.76)	0.014	1.69 (1.07–2.66)	0.023
