# Supplementary material for: Long non-coding RNA ATB promotes malignancy of esophageal squamous cell carcinoma by regulating miR-200b/Kindlin-2 axis
Source: Cell Death Dis. 2017 Jun 22;8(6):e2888–. doi: 10.1038/cddis.2017.245 (PMC5520904; doi:10.1038/cddis.2017.245)
Supplement: Supplementary Tables [file cddis2017245x1.docx]

Supplementary Table 1: Gene annotations of lnc-ATB

|  | Characteristics |
| --- | --- |
| Names | lncRNA activated by transforming growth factor β (lnc-ATB)  lncRNA-AL589182.3  ENST00000493038 |
| Location | chr14:19,858,667-19,941,024 |
| Length | 2446 nucleotides |
|  |  |

Supplementary Table 2: shRNA sequences targeting lnc-ATB

| Names | Sequence(5'-3') |
| --- | --- |
| sh#1  sense | GATCCGCCTTATGGCCTAGATTACCTTTCCATTCAAGAGATGGAAAGGTAATCTAGGCCATAAGGCTTTTTTG |
| sh#1  anti-sense | AATTCAAAAAAGCCTTATGGCCTAGATTACCTTTCCATCTCTTGAATGGAAAGGTAATCTAGGCCATAAGGCG |
| sh#2  sense | GATCCGCCTGTCTGTATTTGCGAATACCTTTTTCAAGAGAAAAGGTATTCGCAAATACAGACAGGCTTTTTTG |
| sh#2  anti-sense | AATTCAAAAAAGCCTGTCTGTATTTGCGAATACCTTTTCTCTTGAAAAAGGTATTCGCAAATACAGACAGGCG |
| NC  sense | GATCCGTTCTCCGAACGTGTCACGTTTCAAGAGAACGTGACACGTTCGGAGAACTTTTTTG |
| NC  anti-sense | AATTCAAAAAAGTTCTCCGAACGTGTCACGTTCTCTTGAAACGTGACACGTTCGGAGAACG |

Supplementary Table 3: Primers used for vector construction

| Primer names | Sequence(5'-3') |
| --- | --- |
| pcDNA3.1-ATB-WT forward | CTCAAGCTTGGCCCTGGGGCTCTGCAA |
| pcDNA3.1-ATB-WT reverse | GGAATTCTGGTAAATGAGTCCAAAGTC |
| pcDNA3.1-ATB-Mut  forward | GATCAACAGAGAGTAACAGAGGACTATTGTGTTATTT |
| pcDNA3.1-ATB-Mut reverse | AGTCCTCTGTTACTCTCTGTTGATCGAATCCC |
| pmirGLO-ATB (WT or Mut) forward | CTAGTTGTTTAAACGTGTTTTCCTTTGCTTCCTCT |
| pmirGLO-ATB (WT or Mut) reverse | GCAGGTCGACTCTAGTCATACTGCCCCTCCCG |
