# Supplementary figures and images for: Long non-coding RNA ATB promotes malignancy of esophageal squamous cell carcinoma by regulating miR-200b/Kindlin-2 axis
Source: Cell Death Dis. 2017 Jun 22;8(6):e2888–. doi: 10.1038/cddis.2017.245 (PMC5520904; doi:10.1038/cddis.2017.245)

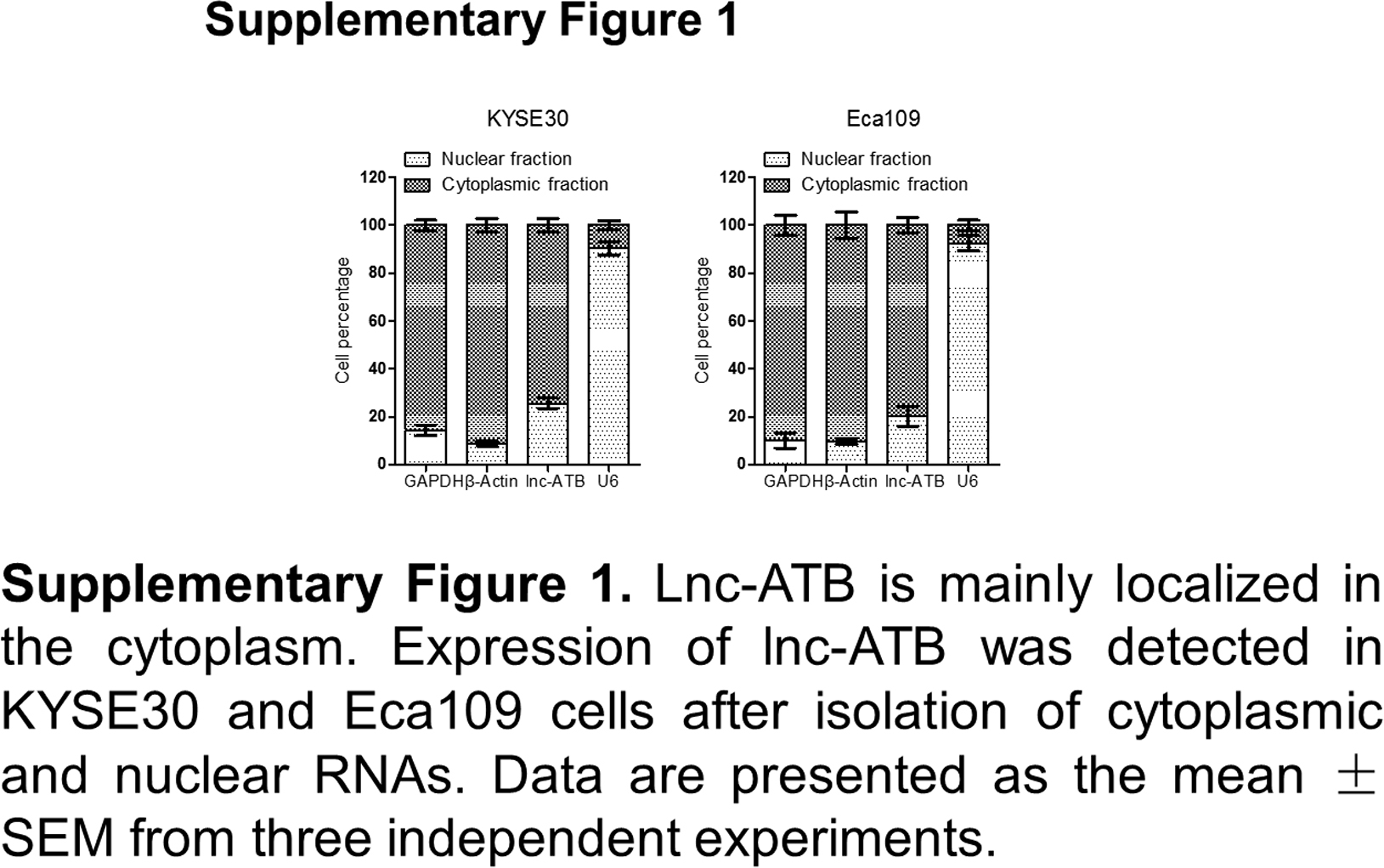

Supplement: Supplementary Figure S1 [file cddis2017245x2.tif]

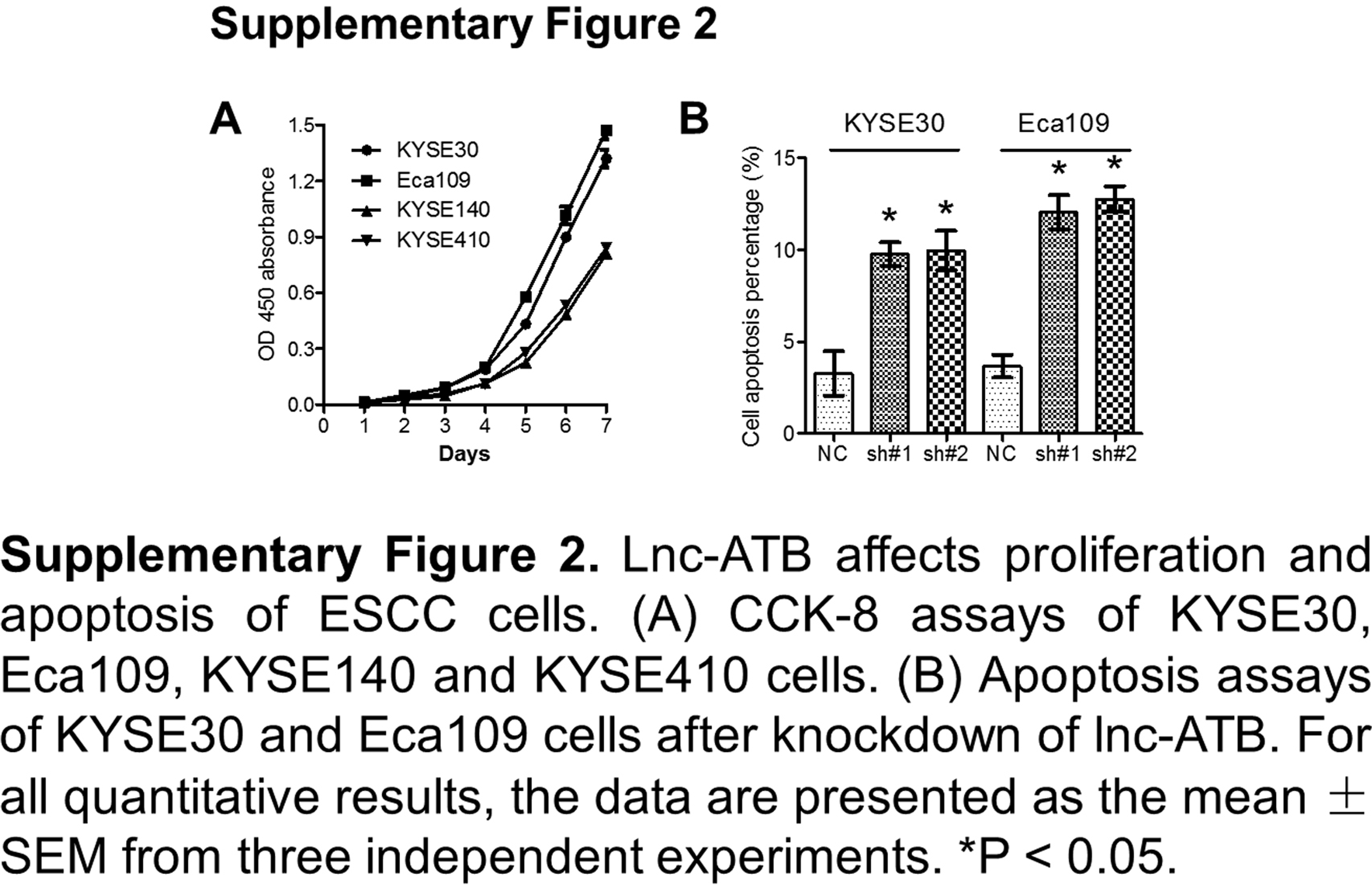

Supplement: Supplementary Figure S2 [file cddis2017245x3.tif]

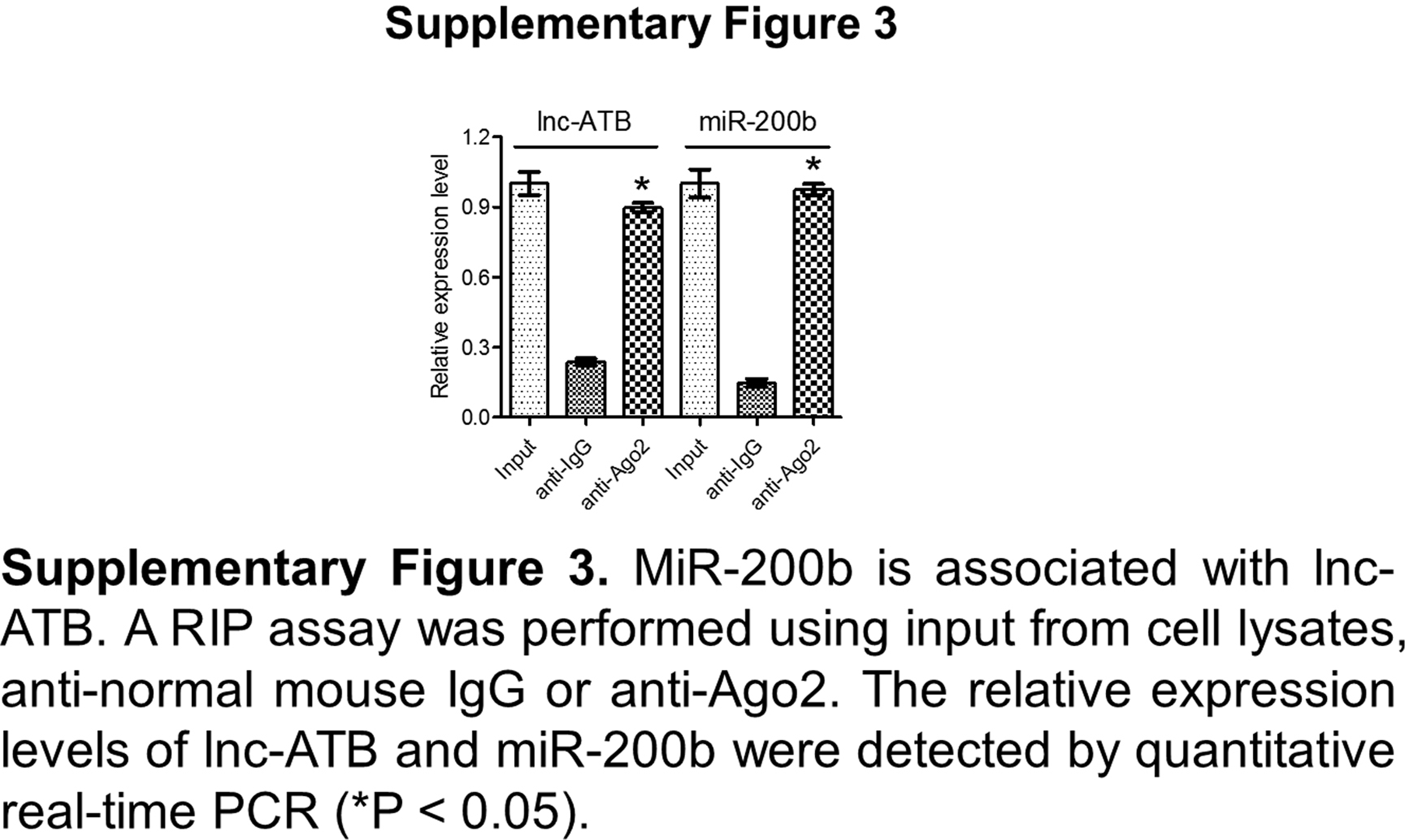

Supplement: Supplementary Figure S3 [file cddis2017245x4.tif]
